# The Association between the Usage of Non-Steroidal Anti-Inflammatory Drugs and Cognitive Status: Analysis of Longitudinal and Cross-Sectional Studies from the Global Alzheimer’s Association Interactive Network and Transcriptomic Data

**DOI:** 10.3390/brainsci10120961

**Published:** 2020-12-10

**Authors:** Robert Morris, Kyle Armbruster, Julianna Silva, Daniel James Widell, Feng Cheng

**Affiliations:** 1 Department of Pharmaceutical Science, College of Pharmacy, University of South Florida, Tampa, FL 33613, USA; rpm4@mail.usf.edu; 2 Biomedical Science Program, College of Art and Science, University of South Florida, Tampa, FL 33620, USA; karmbruster@mail.usf.edu (K.A.); juliannas@mail.usf.edu (J.S.); widell@mail.usf.edu (D.J.W.)

**Keywords:** GAAIN, NSAID, memory, microarray, Alzheimer’s disease, arachidonic acid

## Abstract

The degenerative cognitive and physical decline of Alzheimer patients, coupled with the extensive psychological and economic tolls imposed on family members that serve as caretakers, necessitate the discovery of effective cures and preventative measures for age-related cognitive depreciation. In the journey of Alzheimer’s disease treatment discovery, several cross-sectional and longitudinal studies have delineated a noticeable association between the use of nonsteroidal anti-inflammatory drugs (NSAIDs), a class of low-cost drugs with minimal side effects, and the alleviation of age-related memory impairment. In this study, four datasets (two cross-sectional and two longitudinal studies) derived from the Global Alzheimer’s Association Interactive Network (GAAIN) were analyzed. The significant association between the usage of NSAIDs and better cognitive status was observed. The results agree with the findings of previous studies that the use of NSAIDs may be beneficial in the early stages of Alzheimer’s disease. Transcriptomic data show that ibuprofen treatment results in upregulation of several genes involved in arachidonic acid metabolism including PPARγ, Cyp4a12b, Cyp2c66, and Cyp2c37 in the hippocampus. The increase in conversion of arachidonic acid into anti-inflammatory 16C and 18C dicarboxylic acids as well as epoxyeicosatrienoic acids may play a role in reducing the risk of Alzheimer’s disease development.

## 1. Introduction

Alzheimer’s disease (AD) is a neurodegenerative disorder that leads to severe cognitive decline due to neuronal degradation and subsequent accumulation of plaques and neurofibrillary tangles [1,2,3]. The progressive atrophy of the temporal lobe and frontal cortex, among other regions of the brain which are degraded, results in loss of memory function as well as disorientation and alterations in normal psychological behavior [1,2,3]. Approximately 6 million Americans are impacted by various stages of AD-associated cognitive decline, the majority of whom are patients over the age of 65, and AD contributes to approximately 70% of all clinically diagnosed cases of dementia globally [3]. The extensive long-term care required for impacted patients, coupled with a lack of available treatments and ineffective non-specific palliative care, generates a substantial economic burden and emphasizes the need for new therapeutic discoveries. 

Nonsteroidal anti-inflammatory drugs (NSAIDs) are a class of drugs that are frequently used to alleviate pain and reduce systemic and localized inflammation through the inhibition of cyclooxygenase (COX) activity and the subsequent decline of pro-inflammatory prostaglandins [4,5]. The over-the-counter nature and accessibility of these pharmaceuticals allows for an inexpensive means for the treatment of some diseases. When administered in low doses (75–160 mg/day), aspirin primarily inhibits the constitutively active COX-1 and modulates the conversion of arachidonic acid into pro-inflammatory prostaglandins, which regulates the aggregation of platelets and reduces the formation of blood clots [5]. High dose regimens of aspirin (>325 mg) results in greater inhibition of COX-2, the inducible isoenzyme that promotes the synthesis of pro-inflammatory prostaglandins and clotting factors such as thromboxane upon infection or wounding [5]. 

Some studies have implicated common NSAIDs such as ibuprofen and aspirin in the alleviation of symptoms in patients with early-stage AD [1,2,3]. The purpose of this study is to demonstrate whether NSAID use is associated with cognitive function using longitudinal and cross-sectional studies from the Global Alzheimer’s Association Interactive Network (GAAIN, http://www.gaain.org/) [6,7,8], the largest online integrated research platform. GAAIN comprises an extensive database of 51 AD cross-sectional and longitudinal studies from across the globe, of which the Alzheimer’s Disease Repository Without Borders (ARWIBO), the Kame Project (KAME), Arizona APOE4 Gene Dose Program (APOE4), and Long Beach Longitudinal Study (LPLS) datasets were utilized for this study to investigate the effects of NSAIDs on cognition status. Transcriptome data obtained from microarray [9,10,11] or RNA-seq [12] are often used to gain insight into the molecular mechanism of medications [13,14]. Our analysis of a microarray dataset shows enriched genes and associated pathways in an Alzheimer’s disease mouse model on an ibuprofen regimen, which may explain the association at the molecular level.

## 2. Experimental Section

Two longitudinal and two cross-sectional studies were selected from the GAAIN database. A cross-sectional study collected data from a population at one specific time point. A longitudinal study is a research design that involves repeated observations of variables over a period of time. Three statistical models were provided in the GAAIN analysis module: logistic regression models, linear regression models, and COX proportional-hazard models. Logistic regression models are typically utilized for categorical outcomes, while linear regression models and COX proportional-hazard models are used for continuous outcomes and survival analysis, respectively. Utilizing the provided GAAIN statistical tools, *p*-values and odds ratios were determined for comparing people taking an NSAID with those not taking. A *p*-value < 0.05 was identified as a statistically significant association for all 4 studies.

The ARWIBO dataset is a cross-sectional study that includes data from people in Brescia and the surrounding areas of Northern Italy. Test data include PET scan images and structural images derived from healthy elderly controls, individuals with mild cognitive impairment, and people with Alzheimer’s disease. 

The KAME longitudinal study examined risk factors associated with dementia and various cognitive subtypes in Japanese immigrants that migrated to Seattle. Cognitive status of the phase 3 (P3MMSE) KAME study was measured using the standard protocol in which a value = 30 represents healthy cognitive function, a value = 25 represents MCI, and a value < 20 represents severe dementia-associated impairment. Odds ratios and *p*-values were determined by a logistic regression model included in the GAAIN statistical tool. 

The APOE4 longitudinal study performed by Mayo Scottsdale and Banner Alzheimer’s Institute in Arizona, USA examined various biomarkers and cognitive status every two years for people with either 2, 1, or 0 copies of the APOE4 allele. Immediate (short-term) memory and delayed (long-term) memory were tested using the standard Logical Memory Test. A simple story (Story A) was read to each people and the people was subsequently asked to recall details from the story either immediately after the story was read for immediate memory or 20 min after for delayed memory. A value ranging from 0 and 25 was then assigned to each person to determine strength of memory with higher values reflecting greater recall of the story. 

Finally, the LBLS dataset analyzed the cognitive change in people from the Long Beach and Orange County, California, USA ranging in age from 28–103 and measured the fluctuations in cognitive function over a 15-year period. For immediate working memory, the revised Neo Personality Inventory (NEO-PI-R) was utilized, a test of 240 items that measures the 5 major aspects of personality including neuroticism, agreeableness, conscientiousness, extraversion, and openness. Each aspect contains 6 subgroups, known as facets, are each scored from 0–5 and totaled together at the end. To determine the strength of a people’s ability to internalize and recall language, the standard protocol for the Schaie–Thurstone Test of Mental Ability (STAMAT) was adhered to. For immediate recall, a series of 20 words were spoken over a 3.5 min period and people were asked to recall the words in any particular order immediately preceding the 3.5 min period. For delayed recall, people were administered additional psychometric tests for 1 h, and then asked to recall words from the list in any order.

In addition, transcriptomic data derived from National Center for Biotechnology Information (NCBI) Gene Expression Omnibus (GEO) database [15,16] (GEO ID: GSE67306 [17]) was analyzed in order to determine the effects of ibuprofen on the transcriptomic profile of the hippocampus of APP-PS1 mice, a commonly used double transgenic Alzheimer’s mouse model. A list of statistically significant genes was compiled using the GEO2R [16] data tool by comparing the transcriptomes of 5 mice that took a daily dose of 375 ppm ibuprofen to 5 control mice that were not administered ibuprofen. For upregulated and downregulated genes in the APP-PS1 vs. control, the cutoff for significance was *p* < 0.05 and the absolute value of log_2_(fold change) > 0.15. The generated gene lists were then submitted into DAVID Functional Annotation Bioinformatics Microarray Analysis web server (https://david.ncifcrf.gov/) in order to delineate enriched mechanistic pathways in the hippocampus of APP-PSA mice [18,19,20]. Pathways generated by both BIOCARTA and KEGG [21] were analyzed.

## 3. Results

### 3.1. ARWIBO Dataset

Results of ARWIBO dataset indicate that there is a significant association (*p*-value = 0.01) between taking NSAIDs and cognitive status. As shown in Table 1, a total of 214 people were tested for cognitive status, of which 184 individual were not taking NSAID medication (the control group) and 30 of which were taking NSAIDs (the NSAID group). Of the 184 people in the control group, 121 were female and 63 identified as male. In the NSAID group, the sex distribution was 20 females and 10 males. Control and NSAID groups showed no significant difference with respect to age or sex as evident by the *p*-values = 0.82 and 0.80, respectively. In the control group, 108 people demonstrated dementia while 76 people exhibited mild cognitive impairment (MCI). In the NSAID group, 10 people were diagnosed with dementia while 20 people have MCI. A significant association (*p*-value = 0.01) between taking NSAIDs and cognitive status was identified. The NSAID group was less likely to have dementia than the control group.

### 3.2. KAME Dataset

The effects of aspirin on the Mini-Mental State Examination (MMSE) score, a widely used assessment of cognitive function, were identified from the KAME dataset derived from the GAAIN database. The dataset investigated the risk factors and prevalence of Alzheimer’s disease among the Japanese American population of Seattle, WA. As shown in Table 2, there is a significant association (*p*-value = 0.01) between taking aspirin and MMSE. Of the 286 people analyzed, 233 were part of the control group and 53 were taking aspirin. The sex distribution for the control group was 148 females and 85 males, while the aspirin group comprised 27 females and 26 males. Age and sex demonstrated no significant difference between two groups (*p*-value = 0.17 and 0.10), while higher cognitive score was observed in the aspirin group. The average cognitive score for the control group was 20.19, while the corresponding mean cognitive score value for the aspirin group was 22.68. 

### 3.3. APOE4 Dataset

The effects of NSAID use on both immediate (short-term) and delayed (long-term) memory was assessed from the Arizona APOE4 Gene Dose Program (APOE4) derived from the GAAIN database. As indicated in Table 3, there was a statistically significant association (*p* = 0.02) between the use of an NSAID regimen and the resulting WMS-r score for immediate memory recall. Of the 489 people analyzed, 300 people did not take any NSAIDs, while 189 individuals were on an active NSAID regimen. The sex distribution for the control group included 228 females and 72 males, while the distribution of the NSAID group comprised 152 females and 37 males. Sex did not demonstrate a statistically significant difference between these two groups (*p* = 0.40). Age demonstrated a significant difference between the control group and the NSAID group (*p* = 0.05) with an odds ratio of 1.02. The average age of people in the NSAID group was approximately 2 years older than those in the control group. 

Despite this age difference, memory was still stronger in the NSAID group when compared to the control group. There was also a significant association between the utilization of NSAIDs and the logistic regression of immediate memory (*p* = 0.04) with an odds ratio of 1.07. In terms of WMS-r, the average score for immediate memory recall increased from 13.68 to 14.27 in the NSAID group. The efficacy of NSAID on delayed memory was also assessed. There was a statistically significant association between the use of NSAIDs and the resulting WMS-R score for delayed memory recall (*p* = 0.01). The odds ratio for delayed memory was 1.06.

### 3.4. LBLS Dataset

The association between a daily aspirin regimen and improvement in cognitive function was analyzed using the LBLS study, a 15-year longitudinal project that analyzed the age-related changes in cognitive function in people aged 28–103. Of the 1414 people analyzed in this study, 627 members comprised the control group and were not frequent users of aspirin or other NSAIDs, while 787 people took a daily low-dose regimen of aspirin. In the control group, 323 people were female, and 304 people were male. The NSAID group comprised 461 females and 326 males. As indicated by Table 4, age and sex were not statistically significant different between these two groups with *p*-values of 0.07 and 0.06. However, there was a significant association between aspirin use and the resulting strength of immediate memory (*p* = 0.003) as measured by the NEO-PI-R psychometric test with an average of 0.59 for people who did not take aspirin and an average of 0.64 for people on a lose dose daily regimen. This particular relationship had a odds ratio of 2.38. The statistically significant interrelation between NSAID use and verbal recall was also observed for STAMAT-measured immediate and delayed recall of verbally given information as indicated by Table 5. For immediate recall, the average number of recalled words increased from 5.01 to 5.4 in people undergoing an aspirin regimen when compared with the control had an associated *p*-value of 0.01, and an odds ratio of 1.21 in people who take aspirin daily. For delayed recall, the average number of recalled words in the control group was 3.43 and was 3.87 in the aspirin group. The associated *p*-value and odds ratio were 0.003 and 1.18, respectively.

### 3.5. Transcriptomic Data Analysis

In the APP-PS1 model, 679 gene probes were found to be upregulated in the mice administered a 375 ppm dose of ibuprofen when compared to the control mice who were not administered ibuprofen. For downregulated genes, 909 gene probes were found to be differentially expressed in the ibuprofen-administered mice relative to the control. Table 6 shows the statistically significant KEGG and BIOCARTA pathways (*p* ≤ 0.05) for the upregulated genes and the gene count for each pathway. 

**Figure 1:**
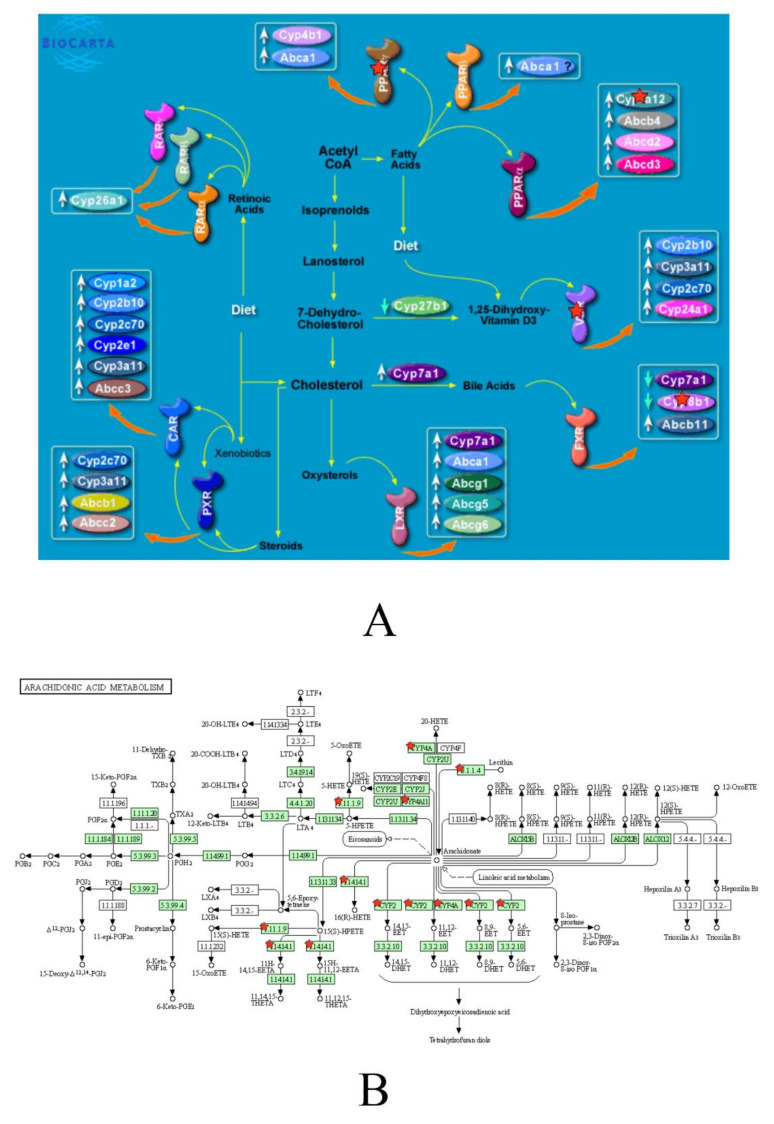
Two significant pathways identified from upregulated genes in ibuprofen-treated APP-PS1 mice (**A**) “Nuclear receptors in lipid metabolism and toxicity” from BIOCARTA pathways (**B**) “Arachidonic acid metabolism” from KEGG pathways. Upregulated genes identified from microarray are marked with a red star.

BIOCARTA pathway analysis generated an enriched list of four upregulated genes in the APP-PS1 mouse treated with ibuprofen and were mapped to a pathway involving the metabolism of fatty acids (nuclear receptors in lipid metabolism and toxicity pathway). As indicated in Figure 1A, the aforementioned upregulated genes included Cyp4a12b, Cyp8b1, peroxisome proliferator activated receptor gamma (PPARγ), and vitamin D receptor (Vdr). KEGG pathway analysis generated a list of seven upregulated genes in the APP-PS1 mouse model and were mapped to a pathway involving the metabolism of arachidonic acid. As indicated by Figure 1B, upregulated genes in the KEGG analysis included additional members of the cytochrome p450 family such as Cyp2b19, Cyp2c66, Cyp2c37, Cyp4a12b, Cyb4a31, glutathione peroxidase 2 (Gpx2), phospholipase A2, and group IID (Pla2g2d). 

## 4. Discussion

This collective study illustrated a statistically significant association between the NSAID usage and improved cognitive function and recollection as measured by a series of psychometric examinations including the MMSE score, Logical Memory Test, NEO-PI-R, and STAMAT [22,23]. For the ARWIBO study, the odds ratio of 0.36 indicates that people on a daily NSAID regimen were 64% less likely to have dementia than a person in the control group that was not on an NSAID regimen. In the KAME study, which focused on the memory capacity of people, the odds ratio of 1.08 indicated that people on a low dose of aspirin were 8% more likely to have a higher MMSE score on average, thus higher memory capabilities, than people in the control group. The odds ratio of 2.38 for the APOE4 dataset indicates that there is a 138% stronger association between taking NSAIDs daily and scoring higher on the NEO-PI-R examination. For the LBLS longitudinal study, the odds ratio of 1.21 indicates a 21% higher association between NSAID use and greater immediate recall of a list of words. The odds ratio of 1.18 for delayed recall of a list of words demonstrates a comparable correlative relationship with an 18% greater degree of association between NSAID utilization and stronger delayed memory.

In many AD patients, chronic pain arises from overstimulation of microglial cells, the resident macrophages of the central nervous system that can promote various proinflammatory pathways [24]. Dysregulation of norepinephrine-mediated signaling, which has been shown to modulate the pain response of the brain, is thought to disrupt microglial activity and promote their overactivity, thus giving rise to chronic pain [24]. The proinflammatory activation of microglial cells has also been implicated in driving the two primary domains of AD pathology [24], Aβ peptide accumulation and formation of neurofibrillary fibers comprised hyperphosphorylated tau proteins [24]. This driving of AD pathology by overstimulated microglial cells may also provide an explanation for the association between chronic pain and lower cognitive function. One study showed a direct relationship between the degree of chronic lower back pain, as measured by the McGill Pain Questionnaire Short Form, and subsequent cognitive ability (*p* < 0.001) [25]. Neuropsychological testing, particularly the Repeatable Battery for the Assessment of Neuropsychological Status and the Trail Making Test, measured that an increase in chronic pain intensity was associated with lower cognitive function such as impaired short-term memory, impaired delayed memory, and language skills [25]. Thus, an increase in chronic pain severity may work concurrently with the components of AD pathology to promote the decline in cognitive function. From a physiological perspective, frequent NSAID use may reduce inflammation in brain tissue and promote vasodilation of blood vessels. This increase in cerebral blood flow (CBF) and the supplementation of the frontal load with nutrients and oxygen subsequently result in a greater degree of neuronal activation and synaptic function [26].

From a mechanistic approach, some studies have demonstrated that aspirin and other NSAIDs serve as agonists for the nuclear receptors peroxisome proliferator-activated receptor alpha (PPARα) and peroxisome proliferator-activated receptor gamma (PPARγ) [27,28]. Stimulation of cytosolic PPARα by aspirin binding results in the dimerization of PPARα and retinoid X receptor gamma (RXRγ) and subsequent translocation to the nucleus [27,28]. Once in the nucleus, the dimer associates with PPARG coactivator 1 alpha (PGC1α) and binds to the promoter region of the transcription factor EB (TFEB) gene [28,29]. Production of unphosphorylated TFEB, a master regulator of lysosomal biogenesis, promotes both a positive feedback loop that leads to production of more TFEB as well as promotes the expression of essential lysosomal components [28,29,30]. The increase in lysosome production and activity may increase the clearing of accumulated Tau proteins and decrease the entanglement of neurofibrillary fibers, a hallmark of AD, through the autophagy-lysosome pathway (ALP) [29,30]. Concurrently, aspirin also binds to and stimulates PPARγ, which subsequently dimerizes with RXRγ and associates with PGC1α in the nucleus to form a functional transcription factor that stimulates lipid metabolism [27,31]. Working in conjunction with PPARα, PPARγ inhibits the activation of COX-2 through the blocking of pro-inflammatory cytokines such as IL-1β and promotes fatty acid catabolism as opposed to storage of fatty acids [27,31]. As a result, the decrease in production of pro-inflammatory lipids such as prostaglandins and leukotrienes leads to a reduction in localized inflammation and an increase in cognitive function. 

Based on the transcriptomic profile analysis of APP-PS1 mice, stimulation of PPARγ and subsequent metabolic regulation of arachidonic acid is suggested to be the primary mechanism through which NSAID use promotes greater cognitive function. Plag2d2 enzymatically converts membrane-bound phospholipids, commonly linoleic acid, into arachidonic acid, a 20-carbon molecule of which serves as the precursor for a variety of fatty acid metabolites that play a role in the inflammatory response [32,33]. In the absence of an NSAID, arachidonic acid serves as a substrate for both COX-1 and COX-2, resulting in the production of thromboxane A2 (TXA2) and pro-inflammatory prostaglandins such as PGE2, respectively [4,32,33]. TXA2 production by COX-1 increases platelet aggregation, improves blood clotting, and slows the flow of blood to the site through vasoconstriction. In the presence of an NSAID such as ibuprofen, COX-1-mediated clotting is inhibited and blood flow is increased through vasodilation [32,33]. In addition, the cyclooxygenase activity of COX-2 is inhibited and production of PGE2 declines. Ibuprofen and arachidonic acid serve as agonists for the transcription factor PPARγ and promote its localization to the nucleus [31,32,33]. Downstream target genes include a variety of different members of the cytochrome p450 family of enzymes that ultimately convert additional arachidonic acid into a multitude of downstream metabolites. 

**Figure 2:**
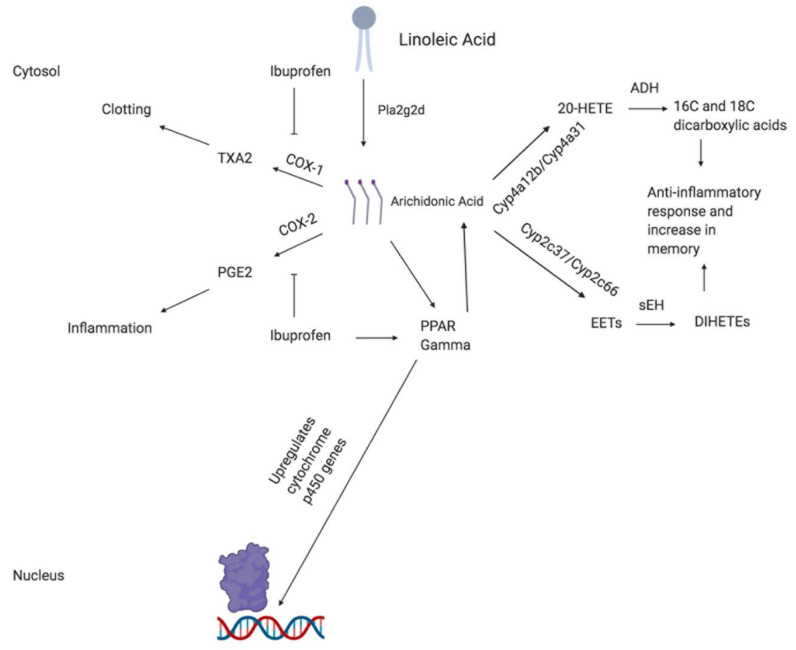
Proposed mechanism outline for the effects of NSAID use on memory. Abbreviation: 20-HETE: eicosanoid 20-hydroxyeicosatetraenoic acid; ADH: alcohol dehydrogenase; DiHETEs: dihydroxyeicosatrienoic acids; EETs: epoxyeicosatrienoic acids; PPAR: peroxisome proliferator activated receptor; COX: cyclooxygenase; Pla2g2d: phospholipase A2, and group IID; sEH: soluble epoxide hydroxylases; TXA2: Thromboxane A2.

With cyclooxygenase activity inhibited, arachidonic acid can then progress through two different metabolic pathways, the choice of which is determined by the monooxygenase (cytochrome p450 family member) that utilizes arachidonic acid as a substrate [34]. In the first pathway, upregulation of Cyp4a12b and Cyp4a31 directs synthesis of the proinflammatory eicosanoid 20-hydroxyeicosatetraenoic acid (20-HETE) through ω-hydroxylation [32,34]. However, subsequent inactivation of 20-HETE by alcohol dehydrogenase (ADH), coupled with β-oxidation, results in the synthesis of anti-inflammatory 16C and 18C dicarboxylic acids. If COX-2 was not inactivated, 20-HETE would instead be converted into the proinflammatory 20-hydroxy-prostaglandin (20-OH-PGE) [32,34]. The second metabolic pathway that arachidonic acid can progress through is mediated by the CYP2C family of cytochrome p450 enzymes. Upregulation of Cyp2c37 and Cyp2c66 in this study promotes conversion of arachidonic acid into epoxyeicosatrienoic acids (EETs). Proceeding inactivation of EETs by soluble epoxide hydroxylases (sEH) yields anti-inflammatory dihydroxyeicosatrienoic acids (DiHETEs) [32,34]. Finally, the upregulation of GP2 helps prevent damage caused by reactive oxygen species (ROSs) that are generated as byproducts during arachidonic acid metabolism. Figure 2 displays an outline of the mechanism proposed for NSAID-induced memory improvement.

Although these preliminary clinical and transcriptomic studies illustrate a promising connection between NSAID use and improved cognitive status, there are some inherent limitations that prompt further research. First, these findings only suggest a significant association between NSAID usage and improved cognitive function as opposed to a causative relationship. Second, although the transcriptomic analysis provides some solid evidence of this association, the translation of mice cytochrome p450 studies into human applications may not be without complications. The relatively small sample sizes of the transcriptome study analyzed limits the applicability of these findings to some degree. In addition, the existing GAAIN tools are not as useful for analyzing the interactions between confounding variables. To solve this problem, new algorithms will be implemented to facilitate this multivariate analysis. Further studies will be conducted through pooled datasets that are formed from GAAIN interrogator tools to increase the size of *n* > 5000 people to obtain more reliable conclusions. In addition, future studies will be performed in order to analyze the difference in efficacy between oral administration and intranasal exposure to various NSAIDs such as ibuprofen, aspirin, and acetaminophen [35,36]. Due to the proximity of the olfactory bulb to the hippocampus, the origin of most cases of AD-related cognitive decline, intranasal administration of NSAIDs may increase the available concentration to the hippocampal region of the brain and enhance the efficacy of NSAIDs in the delaying of cognitive decline onset [35,36]. 

## 5. Conclusions

Four datasets, ARWIBO, KAME, APOE4, and LBLS, in the GAAIN database were analyzed in order to determine the association between NSAIDs on the cognitive status. There was a statistically significant association between improved cognitive function, as measured by a variety of psychometric tests, including the Logical Memory Test, NEO-PI-R, MMSE, and STAMAT, and NSAID usage in people that took NSAIDs on a frequent basis. The results agree with the findings of previous studies that use of NSAIDs may be beneficial in the early stages of Alzheimer’s disease [1,2,3]. The anti-inflammatory nature of NSAIDs may reduce brain swelling and inflammation induced by traumatic injury and, thus, reduce the rate of neuronal cell destruction through stimulation of the lipid metabolism regulators PPARα and PPARγ. The upregulation of cytochrome p450 genes promotes the decrease in pro-inflammatory fatty acids such as PGE2 and an increase in anti-inflammatory signaling molecules such as diHETEs and dicarboxylic acids, thus providing a mechanism in which ibuprofen, and potentially other NSAIDs, may alleviate AD-associated brain inflammation. Despite these promising results, the relatively small sample size of the transcriptome and differences among species are limitations that prevents a more conclusive finding from being drawn. Thus, further large-scale transcriptome study analysis will need to be performed in order to further validate proposed pathways. 

## Figures and Tables

**Table 1:** The analysis results of the Alzheimer’s Disease Repository Without Borders (ARWIBO) dataset using the logistic regression model in Global Alzheimer’s Association Interactive Network (GAAIN) statistical tools. The significant association (*p*-value = 0.01) between taking nonsteroidal anti-inflammatory drugs (NSAIDs) and cognitive status (Dementia or mild cognitive impairment (MCI)) was identified.

	Control Group	NSAID Group	Odds Ratio	*p*-Value
*N*	184	30		
Age	72.14	70.94	0.99 (0.95 to 1.04)	0.82
Sex	121 females 63 males	20 females 10 males	0.90 (0.39 to 2.07)	0.80
Diagnosis	76 (MCI) 108 (Dementia)	20 (MCI) 10 (Dementia)	0.36 (0.16 to 0.82)	0.01

**Table 2:** The statistical results of the Kame project (KAME) data set using the GAAIN tool. The significant association (*p*-value = 0.01) between taking aspirin and cognitive status (the Mini-Mental State Examination (MMSE) score) in the population was identified.

	Control Group	Aspirin Group	Odds Ratio	*p*-Value
*N*	233	53		
Age	81.75	81.86	1.03 (0.99 to 1.06)	0.17
Sex	148 females 85 males	27 females 26 males	1.68 (0.9 to 3.11)	0.10
MMSE	20.19	22.68	1.08 (1.01 to 1.14)	0.01

**Table 3:** The statistical results of using the GAAIN tool for the Arizona APOE4 Gene Dose Program (APOE4) dataset. The significant association (*p*-value = 0.04) between taking NSAID and short-term memory in the population was identified. There was also a significant association between the use of daily NSAID (*p*-value = 0.01) and improved delayed memory recall. Higher assigned values utilizing the Logical Memory Test are indicative of stronger cognitive performance.

	Control Group	NSAID Group	Odds Ratio	*p*-Value
*N*	300	189		
Age	55.47	57.04	1.02 (1.00 to 1.04)	0.05
Sex	228 females 72 males	152 females 37 males	1.22 (0.77 to 1.92)	0.40
Log-mem immed	13.68	14.27	1.07 (1.01 to 1.13)	0.04
Log-mem delayed	12.45	13.22	1.06 (1.01 to 1.11)	0.01

**Table 4:** The statistical results of using the GAAIN tool for the Long Beach Longitudinal Study (LPLS) dataset. The significant association (*p*-value = 0.003) between people taking an NSAID and immediate memory recall, as measured by the Revised NEO Personality Inventory (NEO-PI-R) personality assessment, in the population was identified.

	Control Group	Aspirin Group	Odds Ratio	*p*-Value
*N*	627	787		
Age	70.07	67.24	0.99 (0.98 to 1.00)	0.07
Sex	323 females 304 males	461 females 326 males	1.23 (1.20 to 3.86)	0.06
PI-R Immed.	0.59	0.64	2.38 (1.34 to 4.23)	0.003

**Table 5:** Statistical results derived from the Long Beach Longitudinal Study (LPLS) longitudinal study. The significant association (*p*-value = 0.003) between people taking a daily low-dose regimen of aspirin and immediate memory recall of a list of 20 words (IR (ST)), as measured by the Schaie Thurstone Test of Adult Mental Abilities (STAMAT), in the population was identified. There was also a statistically significant association (*p*-value = 0.003) between daily aspirin use and the delayed recall of total words from an identical list of 20 words (DR (ST)).

	Control Group	Aspirin Group	Odds Ratio	*p*-Value
*N*	115	266		
Age	64.88	65.54	1.01 (0.99 to 1.04)	0.16
Sex	60 females 55 males	161 females 105 males	1.28 (0.81 to 2.02)	0.29
Total words IR (ST)	5.01	5.4	1.21 (1.04 to 1.41)	0.01
Total words DR (ST)	3.43	3.87	1.18 (1.04 to 1.35)	0.003

**Table 6:** Statistically significant (*p*-value ≤ 0.05) pathways identified from upregulated gene probes in ibuprofen-treated APP-PS1 mice. The APP-PS1 is a transgenic mouse model containing a chimeric amyloid precursor protein (APP) and a mutant presenilin 1 (PSEN1).

Pathway Source	Name of Pathway	Gene Count	*p*-Value
KEGG	Olfactory transduction	46	1.2 × 10^−7^
KEGG	Cytokine–cytokine receptor interactions	12	6.2 × 10^−3^
KEGG	Protein digestion and absorption	7	6.4 × 10^−3^
KEGG	Arachidonic acid metabolism	7	6.7 × 10^−3^
KEGG	Janus kinases (JAK)-signal transducer and activator of transcription proteins (STAT) signaling	8	2.0 × 10^−2^
KEGG	Hematopoietic cell lineage	6	2.2 × 10^−2^
KEGG	Retinol metabolism	6	2.7 × 10^−2^
KEGG	Neuroactive ligand–receptor interaction	11	4.4 × 10^−2^
KEGG	Tuberculosis	8	5.0 × 10^−2^
BIOCARTA	Nuclear receptors in lipid metabolism and toxicity	4	2.2 × 10^−2^
BIOCARTA	The co-stimulatory signal during T-cell activation	3	4.6 × 10^−2^
